# GSK-3β inhibition alleviates arthritis pain via reducing spinal mitochondrial reactive oxygen species level and inflammation

**DOI:** 10.1371/journal.pone.0284332

**Published:** 2023-04-14

**Authors:** He-Yu Yang, Xu Sun, Shu-Qing Zhen, Liang-Zhu Yu, Jie-Qiong Ding, Ling Liu, Min Xie, Hai-Li Zhu

**Affiliations:** 1 School of Pharmacy, Xianning Medical College, Hubei University of Science and Technology, Xianning, China; 2 Matang Hospital of Traditional Chinese Medicine, Xianning, China; University of Rijeka Faculty of Medicine: Sveuciliste u Rijeci Medicinski fakultet, CROATIA

## Abstract

Pain is the main symptom of osteoarthritis, which severely reduces the patients’ quality of life. Stimulated neuroinflammation and elevated mitochondrial oxidative stress are associated arthritis pain. In the present study, arthritis model was established by intra-articular injection of complete Freund’s adjuvant (CFA) on mice. Knee swelling, pain hypersensitivity and motor disability were observed in CFA-induced mice. In spinal cord, neuroinflammation was triggered and presented as severe infiltration of inflammatory cells and up-regulated expressions of glial fibrillary acidic protein (GFAP), nuclear factor-kappaB (NF-κB), PYD domains-containing protein 3 (NLRP3), cysteinyl aspartate specific proteinase (caspase-1) and interleukin-1 beta (IL-1β). Mitochondrial function was disrupted and characterized as elevated expressions of B-cell lymphoma 2 (Bcl-2)-associated X protein (Bax), dihydroorotate dehydrogenase (DHODH) and cytochrome C (Cyto C), and reduced expressions of Bcl-2 and Mn-superoxide dismutase (Mn-SOD) activity. Meanwhile, as a potential target for pain management, glycogen synthase kinase-3 beta (GSK-3β) activity was up-regulated in CFA induced mice. To explore potential therapeutic options for arthritis pain, GSK-3β inhibitor TDZD-8 was intraperitoneally injected for three days on CFA mice. Animal behavioral tests found that TDZD-8 treatment elevated mechanical pain sensitivity, suppressed spontaneous pain and recovered motor coordination. Morphological and protein expression analysis indicated that TDZD-8 treatment decreased spinal inflammation score and inflammatory related protein levels, recovered mitochondrial related protein levels, and increased Mn-SOD activity. In summary, TDZD-8 treatment inhibits GSK-3β activity, reduces mitochondrial mediated oxidative stress, suppresses spinal inflammasome response, and alleviates arthritis pain.

## Introduction

Osteoarthritis (OA) is a chronic joint disease, affects mostly middle-aged and older adults. With the aging and increasing life expectancy of the population, OA globally affects about 250 million people, severely reduces the patients’ quality of life and poses a major challenge to social and public health [1]. Pain is the main symptom of OA, especially knee OA, and is described as a dull, aching and unpredicted pain initially, and subsequently more constant over time in OA patients [2]. The common drugs for OA pain management were NASIDs, acetaminophen, and opioid analgesics. Based on the side effects of drugs and the not well-understanding pathogenesis, the current strategy for OA pain relief is not satisfied by patients [3]. Thus, research on the mechanisms of OA pain offers critical new insights for the development of pain treatment.

Central mechanisms and sensitization play an important role in OA pain. Continuous and uncontrolled joint injury or inflammation stimulates nociceptive afferent nerve which endings innervates the joints [4]. These nociceptive signals transmit to the spinal dorsal horn and supraspinal domain, induces central sensitization which presents as amplify pain and reduce threshold, and finally causes the development and maintenance of OA chronic pain [5]. Approximately 5–25% of patients with OA have pain with neuropathic features, which overlap with indicators of centralization [6]. In OA patients, muscle hyperalgesia and extended pain areas are induced by intramuscular infusion of 0.5 ml hypertonic saline [7]. Central sensitization in OA patients has been confirmed by quantitative sensory testing analyses and functional magnetic resonance imaging (MRI) [4, 8, 9]. Accumulating evidences suggest that central sensitization is driven by neuroinflammation, which is characterized by the activation of glial cells, the releasing of pro-inflammatory cytokines and chemokines [10]. In the monosodium iodoacetate (MIA) model of OA joint pain rats, pain behaviors (change in weight bearing and distal allodynia) are assessed, spinal glial cells are activation. Two known inhibitors of glial activation (nimesulide and minocycline) attenuate pain behavior and reduce the number of activated microglia and astrocytes [11]. In collagenase-induced knee osteoarthritis model rats, the development of nociception affects joint and activates of glial cells in the spinal cord. Inhibition of glial cell activation by fluorocitrate decreases these osteoarthritis-associated nociceptive behaviors [12]. Therefore, suppressing neuroinflammation in spinal cord plays a role in the management of chronic OA pain.

Elevated oxidative stress is associated with OA pain. A measurement of 84 patients with chronic osteoarthritis show that higher oxidative stress is associated with greater pain intensity, more widespread pain, greater pain catastrophizing, higher pain interference, and lower function [13]. Mitochondria are the predominant source of intracellular reactive oxygen species (ROS) which is generated by the reaction between oxygen and a little quantity of electrons form electron transport chain (ETC) [14, 15]. Mitochondrial dysfunction and mitochondrial oxidative stress are involved in epigenetic regulation of OA pain [16]. Dichloroacetate administration significantly increases mitochondrial respiratory function and reduces the ipsilateral pain-related behavior in inflammatory pain model rats [17]. The mitochondrial-targeted antioxidant, mitoquinone (MitoQ) treatment inhibits oxidative stress and alleviates vincristine-induced neuropathic pain [18]. Thus, targeting on mt-ROS and inflammation is a potential therapy for neuropathic pain.

Glycogen synthase kinase-3β (GSK-3β) is a potential target for pain management. GSK-3β activity is increased and the mechanical allodynia and thermal hyperalgesia are elevated in the spinal nerve ligation model rats. GSK-3β selective inhibitor administration inhibits GSK-3β activity and decreases the mechanical allodynia [19]. Inhibition of GSK-3β by ghrelin in the spinal dorsal horn markedly alleviates neuropathic pain in a rat model of chronic sciatic nerve constriction injury [20]. Injection of GSK-3β activity inhibitor TDZD-8 suppressed PYD domains-containing protein 3 (NLRP3) inflammasome cascade and consequently decreased mechanical pain sensitivity in cancer-induced bone pain rat model [21]. Pharmacological inhibitors inactivate the GSK‑3β/β‑catenin pathway and attenuate the apoptosis in knee osteoarthritis model [22]. In the current study, in order to clarify the role and pathological mechanism of GSK‑3β on arthritic pain, a complete Freund’s adjuvant (CFA)-induced arthritic pain mouse model was constructed, TDZD-8 was intraperitoneally administered on mice for three consecutive days, and changes in behavioral, morphological and protein expression were analyzed. Our study provides theoretical basis for the mechanism of arthritis pain.

## Materials and methods

### Animals

A total of 27 male C57BL/6J mice weighing 18–20 g (6–8 weeks old) were purchased from Hubei Province Experimental Animal Center (Wuhan, CHN). All animals were housed in a 12h light/dark circumstance with food and water ad libitum. All experimental procedures were performed according to the local and international guidelines on the ethical use of animals, and all efforts were made to minimize the number of animals used and their sufferings. Ethics approval was obtained from the Laboratory Animal Ethics Committee of Hubei University of Science and Technology (2019-03-021).

### Complete Freund’s adjuvant (CFA)-induced arthritis

A modified version of a previously validated mouse model of arthritic inflammation of the knee joint [23]. was produced by performing two intra-articular injections of CFA at days 0 and 7 into the keen joint. Briefly, mice were anesthetized by pentobarbital sodium (60 mg/kg, intraperitoneal injection), followed by an intra-articular injection of CFA (P2036, Shanghai yuanye Bio-Technology, Shanghai, CHN) using a 30-gauge needle that was fitted with cannulation tubing such that only 2.5 mm of the needle was allowed to puncture the left hind joint and ten microliters of CFA was injected into the articular space. The mice in control group were injected with same volume of saline.

### Experimental design

Mice were habituated to the environment for 5 days prior to the experiments, and randomly divided into three groups: Control, CFA and CFA+TDZD-8. Every group contains nine animals. The mice in CFA and CFA+TDZD-8 groups received 10 μl CFA injection on days 0 and 7. At the same time, the mice in control group received equivalent volume vehicle (saline solution). At the 14th day after CFA injection, the mice in CFA+TDZD-8 group received intraperitoneal TDZD-8 (5 mg/kg, S2926, Selleck, Shanghai, CHN) injection for three consecutive days [24]. TDZD-8 was dissolved in DMSO (ST038, Shanghai yuanye Bio-Technology, Shanghai, CHN) and diluted with 0.9% NaCl before used. The control and CFA group received equivalent volume vehicle (DMSO and 0.9% NaCl). Subsequently, behaviors tests were performed at 4 h after TDZD-8 administration. One part of the mice was euthanized with an overdose of pentobarbital sodium (150 mg/kg) by intraperitoneal injection, and then the spinal cords were separated for the ELISA assay and Western blot. The other part was fixed and sectioned for histopathological and immunohistochemical analysis.

### Mechanical threshold test

Mice were placed in a 30 × 30 × 30 cm plexiglass chamber and habituate for at least 30 min before behavioral experiments. The von Frey filaments (Stoelting, Wood Dale, IL, ranging from 0.07 g to 2.0 g) were used by stimulating the left hind paw. Briefly, the filaments were pressed vertically against the plantar surfaces until the filaments were bent and held for 3–5 s. At this situation, a brisk withdrawal and paw flinching was considered as positive response. Once a positive response occurred, the von Frey filament with the next lower force was applied, and whenever a negative response occurred, the filament with the next higher force was applied. Then, the pattern of positive and negative withdrawal response was converted to mechanical threshold [25].

### Spontaneous flinches test

Mice were placed in a 30 × 30 × 30 cm plexiglass chamber and habituated for at least 30 min. The number of flinches was counted sustained 5 min for three times. Take the average of the total number of flinches [26].

### Rotarod test

An accelerating rotarod was used to assess motor coordination and balance of animals (ZS-RDM-XS, Beijing, CHN). Three days before the experiment, the animals accepted acclimatization training at a fixed speed of 4 r/min for 10 min and repeat 3 times at 10-minute intervals. At the beginning of experiment, the rotation speed is set at a fixed value of 10 r/min for 10 s and then accelerate for 10 s. After that, the rod is working at a speed of 20 r/min for 30 s and then accelerate for 10 s. The movement was continuously carried out for 10 min. Repeating three times with an interval of 10 min. The latency to fall of mice was recorded [27].

### H&E staining

After behavioral tests, mice were deeply anesthetized with 60 mg/kg sodium pentobarbital, perfused transcardially with saline containing heparin, following with perfusing to 4% paraformaldehyde (PFA, 0.1 M phosphate buffer, pH 7.4) until the animal body was stiff and rigid. After perfusion, spinal cords were removed and post-fixed in 4% PFA for 12 h at 4°C and embedded in paraffin, and cut into 4-μm sections using a microtome (RM 2165; Leica Microsystems GmbH, Nußloch, GER). The sections were stained using the standard H&E method (H&E staining solution, BL735B, Biosharp Life Sciences, Anhui, CHN). Briefly, sections were treated with xylene, 100% ethanol, 90% ethanol, 70% ethanol for dewaxing, dyed with hematoxylin solution, stained with eosin, sealed with neutral balsam and observed using a fluorescence microscope (IX73, Olympus Corp, Tokyo, JPNp). H&E images were analyzed using the ImageJ 1.51j8 software (National Institutes of Health, US). The scoring criteria of inflammation cell infiltration (26) is: 0 (normal); 1 (lymphocyte infiltration around meninges and blood vessels); 2, 1–10 lymphocytes in a field); 3 (11–100 lymphocytes in a field); 4 (>100 lymphocytes in a field).

### Immunofluorescence analysis

Spinal cord sections were dewaxed, conducted to antigen retrieval (Improved Citrate Antigen Retrieval Solution, P0083, Beyotime Biotechnology, Shanghai, CHN), treated with 3% hydrogen peroxide for 10 min, blocked with immunofluorescence blocking solution (P0102, Beyotime Biotechnology, Shanghai, CHN, room temperature) for 1 h, and then incubated with primary antibody overnight at 4°C, subsequently, incubated with fluorescent secondary antibody at room temperature for 1 h and observed under a fluorescence microscope (IX73, Olympus Corp, Tokyo, JPN). The fluorescence intensities were analyzed using ImageJ 1.51j8 (National Institutes of Health, US). The following primary antibodies were used: anti-IL-1β (1:100, A19635), anti-GFAP (1:100, A0237), anti-NLRP3 (1:100, A5652), anti-caspase-1 (1:100, A0964), anti-Bcl-2 (1:100, A0208) and anti-GSK-3β (1:100, A6164) were from Abclonal (Wuhan, CHN); anti-NF-κB (1:100, BF8005) and anti-DHODH (1:100, DF3991) were from Affinity (Jiangsu, CHN). The secondary antibodies used for immunofluorescence analysis were goat anti-mouse IgG H&L (1:100, FITC, ab6785) and goat anti-rabbit IgG H&L (1:100, FITC, ab6717) were purchased from Abcam (Cambridge, UK).

### Western blotting

After behavioral tests, mice were euthanized with an overdose of pentobarbital sodium (150 mg/kg) by intraperitoneal injection and sacrificed through decapitation. Lumber spinal cord samples were collected, homogenized in RIPA lysis buffer containing 1% protease inhibitors (BBI, Shanghai, CHN), centrifugated at 12,000 g, 4°C for 20 min. Then the supernatant was collected, separated on SDS-PAGE, and transferred to 0.22 μm PVDF membranes. Protein concentration was quantified using a BCA protein assay kit (BL521A, Biosharp Life Sciences, Anhui, CHN). Then the membranes were blocked with QuickBlock^™^ Blocking Buffer for Western Blot (P0023B, Beyotime Biotechnology, Shanghai, CHN), incubated with the appropriate primary antibodies overnight at 4°C. And HRP-conjugated secondary antibodies in TBST (1:5,000) at room temperature for 1 h. Protein bands were visualized using ECL detection reagent (BL520A, Biosharp Life Sciences, Anhui, CHN) and detected with an iBright 1500 instrument (Invitrogen, Thermo Fisher Scientific, Inc). The grey values of bands were analyzed using ImageJ 1.51j8 software (National Institutes of Health, US). β-actin was used as a loading control. The following primary antibodies were used: anti-IL-1β (1:1000, A19635), anti-GFAP (1:1000, A0237), anti-NLRP3 (1:1000, A5652), anti-caspase-1 (1:1000, A0964), anti-Bcl-2 (1:1000, A0208), anti-BAX (1:1000, A0207), anti-Cytochrome C (1:1000, A4912), anti-GSK-3β (1:1000, A6164), and anti-β-actin (1:50000, AC026) were from Abclonal (Wuhan, CHN); anti-Phospho-GSK3β (Ser9) (AF2016), anti-NF-κB (1:1000, BF8005,) and anti-DHODH (1:1000, DF3991) were from Affinity (Jiangsu, CHN). The secondary antibodies used for Western blotting were HRP goat anti-rabbit IgG (H+L) (1:10000, AS014) and HRP goat anti-mouse IgG (H+L) (1:10000, AS003) were purchased from ABclonal Technology.

### Mn-SOD activity detection

For determination of manganese superoxide dismutase (Mn-SOD) enzyme activity, Cu/Zn-SOD and Mn-SOD assay kit with WST-8 (S0103, Beyotime Biotechnology, Shanghai, CHN) was prepared. Briefly, spinal cords were homogenized on ice-cold phosphate buffered saline (PBS) buffer, centrifuged at 12,000 g for 15 min, and the supernatant was collected and incubated with Cu/Zn-SOD inhibitors for 1 h at 37°C. After mixed with WST-8 enzyme working solution for 20 min at 37°C, the OD 450 nm absorbance value of each pore was measured. Mn-SOD activity was expressed as units per milligram of total protein (U/mg protein).

### Mitochondrial membrane potential (MMP) measurement

Mitochondrial membrane potential was measured using fluorescent probe JC-1 (C2003S, Beyotime Biotechnology, shanghai, CHN). Cells were inoculated in the 24-well plate with sterilized coverslips, stimulated with IL-1β (5 ng/ml) for 30min and incubated with 1 μM TDZD-8 for 24 h. Then, cells were cultured with 200 μl JC-1 for 20 min at 37°C in the dark, and the fluorescence intensity was detected by fluorescence microscopy (IX73, Olympus Corp, Tokyo, JPN).

### Mitochondrial ROS measurement

MitoSOX Red mitochondrial superoxide indicator (Invitrogen, Thermo Fisher Scientific, Inc), a novel fluorogenic dye, was used to detect mitochondrial superoxide in cells. Briefly, the cells were inoculated in the 24-well plate with sterilized coverslips, stimulated with IL-1β, incubated with TDZD-8, and subsequently incubated with 5 μM MitoSOX Red (Molecular Probes) in the dark at 37°C for 10 min and detected under fluorescence microscope (IX73, Olympus Corp, Tokyo, JPN).

### Statistical analysis

All statistical analyses were performed using Prism version 8.02 (GraphPad Software, San Diego, CA). One-way analysis of variance (one-way ANOVA) was used to check differences in experimental groups followed by Bonferroni post hoc tests. Results of behavioral tests presented as mean ± SEM. Other experiment data are presented as mean ± SD unless otherwise noted. *P* < 0.05 was considered as the criterion for a significant statistical difference.

## Results

### TDZD-8 treatment relieves pain hypersensitivity on CFA-induced mice

**Fig 1:**
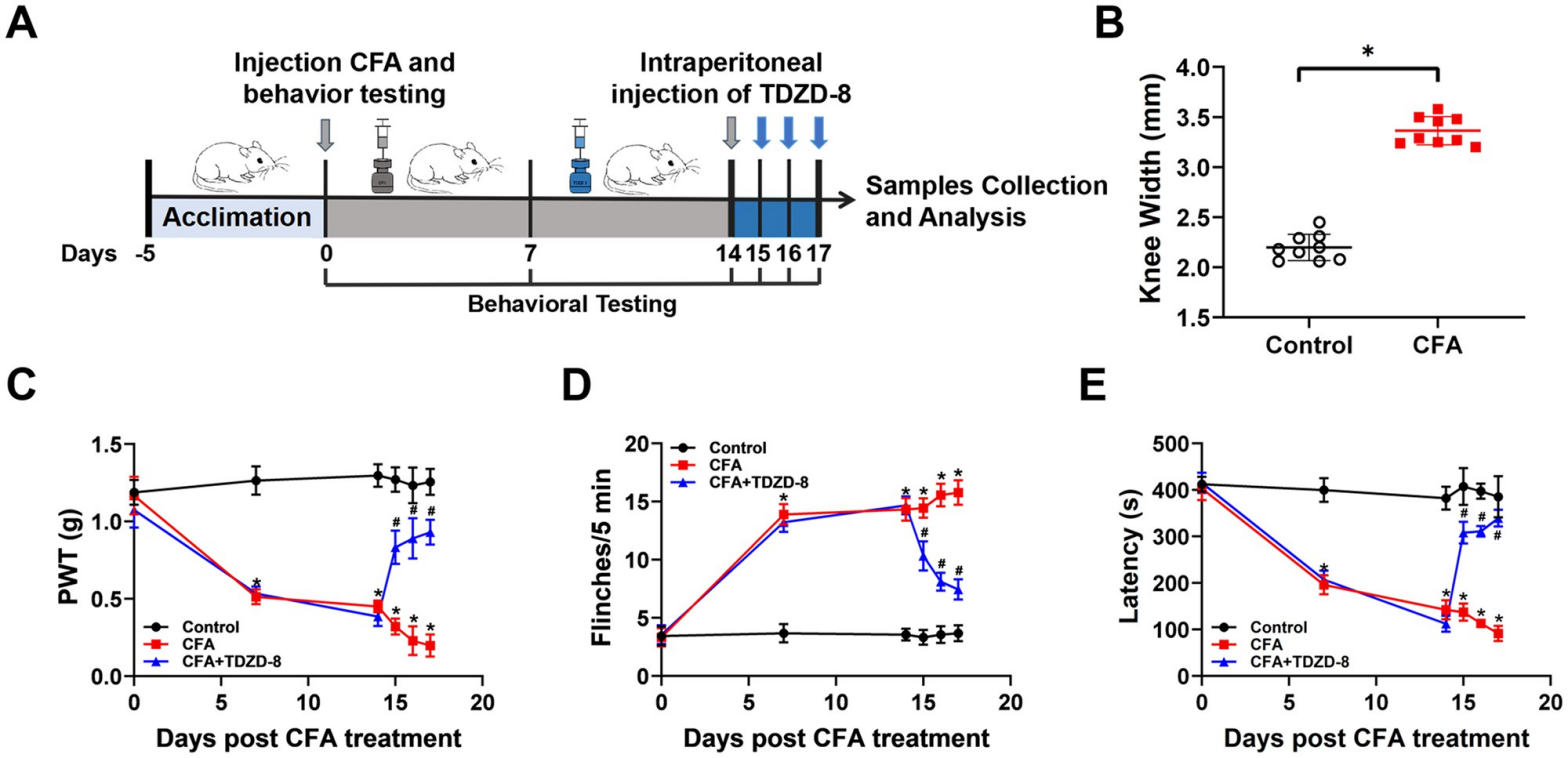
Effect of TDZD-8 treatment on pain behaviors of CFA-induced mice. (A) Schematic diagram of the experimental procedures. On day 0, CFA was intra-articularly injected into the left knee joint of mice, and behavioral tests were conducted at day 0, 7, and 14. TDZD-8 was intraperitoneally injected in mice on day 15, 16 and 17. After 4 h of TDZD-8 treatment, behavioral tests were conducted. Subsequently mice were sacrificed and spinal cord tissues were collected for the further analysis. (B) Changes of knee width of control and CFA mice on day 14 after CFA inducement. Changes of PWT values (C) spontaneous flinches (D) and latency (E) to fall of mice. Data are expressed as the mean ± SEM (n = 9). *P < 0.05 *vs*. control group, ^#^*P* < 0.05 *vs*. CFA group.

Behavioral tests were performed following protocol as shown in Fig 1A. On day 14, CFA inducement leads to knee swelling presenting as increased knee width in CFA group (Fig 1B). Meanwhile, on day 7 and 14 of mice, CFA inducement significantly decreased mechanical threshold values presenting mechanical pain sensitivity (Fig 1C), dramatically increased numbers of flinches presenting spontaneous pain (Fig 1D), also reduced latency to fall presenting motor coordination (Fig 1E). These data revealed that CFA inducement caused pain hypersensitivity and motor disability on mice.

In order to alleviate CFA-induced pain, intraperitoneal administration of TDZD-8 was performed on mice for three consecutive days, and the effect of TDZD-8 on pain sensitivity and motor ability were tested. TDZD-8 treatment statistically raised mechanical threshold values (Fig 1C), obviously descended flinches numbers (Fig 1D), and apparently increased latency to fall on CFA+TDZD-8 mice (Fig 1E). These data illustrated that TDZD-8 treatment reduced mechanical pain sensitivity, suppressed spontaneous pain and recovered motor coordination of CFA-induced arthritis mice.

### TDZD-8 treatment suppresses spinal inflammation

**Fig 2:**
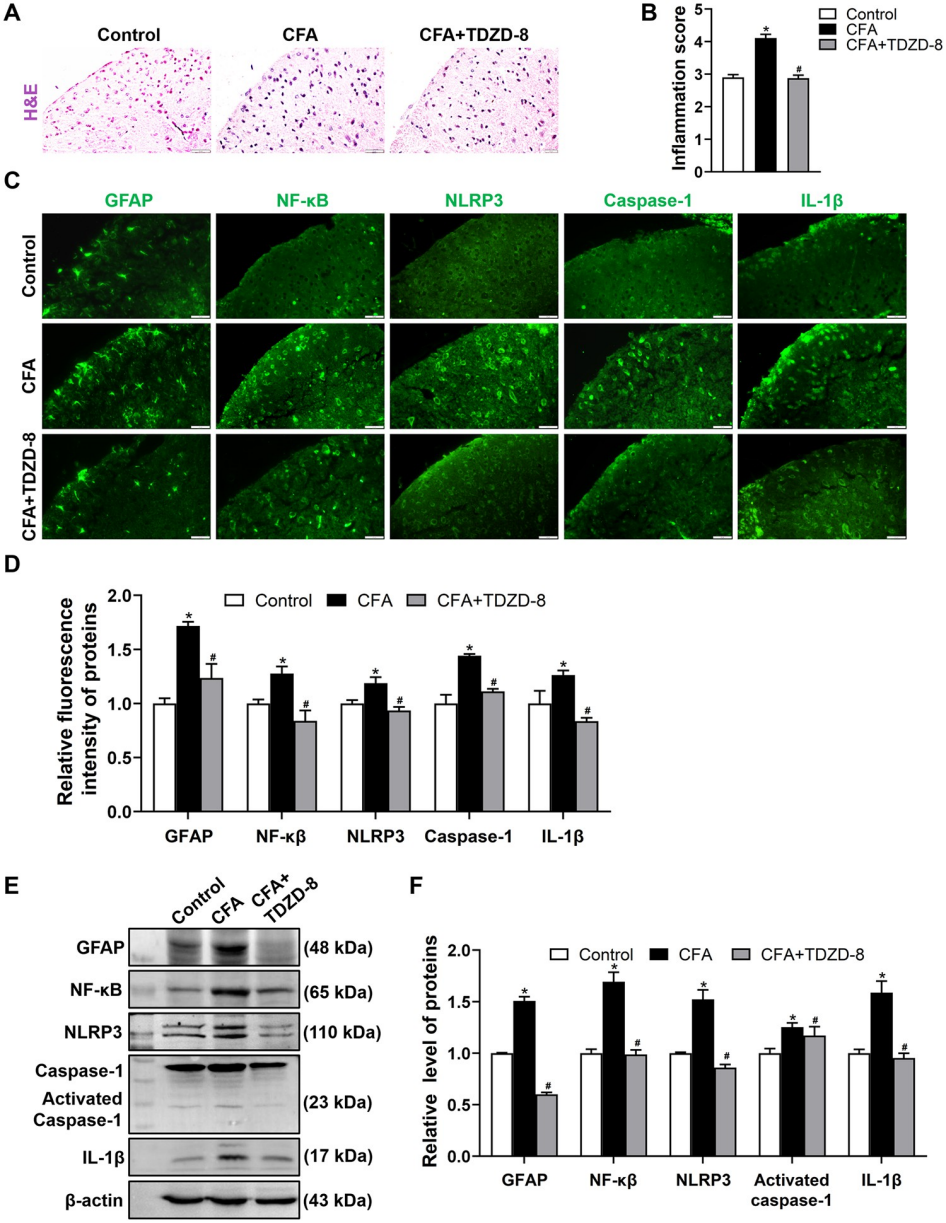
Effects of TDZD-8 treatment on spinal inflammatory infiltration and spinal GFAP, NF-κB, NLRP3, caspase-1 protein levels. (A) Representative H&E staining images of spinal cord sections from control, CFA and CFA + TDZD-8 groups. The red arrows indicated inflammatory cells, scale bar = 20 μm. (B) Quantitative analysis of inflammation score for the H&E staining in each group. (C, D) Representative immunofluorescence staining images of GFAP, NF-κB, NLRP3, caspase-1, IL-1β expressions in spinal dorsal horn (C) and quantitative fluorescence intensity analysis (D), scale bar = 20 μm. (E, F) Western blot analysis (E) and quantification of the relative grey value (F) of GFAP, NF-κB, NLRP3, caspase-1, IL-1β expression level in spinal cord of control, CFA and CFA + TDZD-8 groups. Data are presented as mean ± SD (n = 3). *P < 0.05 *vs*. control group, ^#^*P* < 0.05 *vs*. CFA group.

In spinal dorsal horn of CFA-induced mice, severe infiltration of inflammatory cells was observed detecting by H&E staining (Fig 2A and 2B). Glial fibrillary acidic protein (GFAP) was used as a marker of activation and proliferation of astrocyte which is an important source for inflammation cytokines [28]. The intensity of GFAP in spinal dorsal horn of CFA group was dramatically increased, and TDZD-8 treatment decreased the intensity (Fig 2C and 2D). Consistently, spinal GFAP expression in CFA group was up-regulated, and this up-regulated GFAP expression was reduced by TDZD-8 treatment (Fig 2E and 2F).

Meanwhile, fluorescence intensity of NF-κB, NLRP3, caspase-1, IL-1β in spinal dorsal horn of CFA group was significantly enhanced, in comparison with control group (Fig 2C and 2D). Western blotting showed that the expression levels of spinal NF-κB, NLRP3, caspase-1, IL-1β in CFA group were consistently increased (Fig 2E and 2F). While TDZD-8 treatment decreased the inflammatory response, reduced NF-κB, NLRP3, caspase-1, IL-1β intensity (Fig 2C and 2D), and decreased NF-κB, NLRP3, caspase-1, IL-1β expression levels in CFA+TDZD-8 group (Fig 2E and 2F).

### TDZD-8 treatment inhibits spinal GSK-3β activity

**Fig 3:**
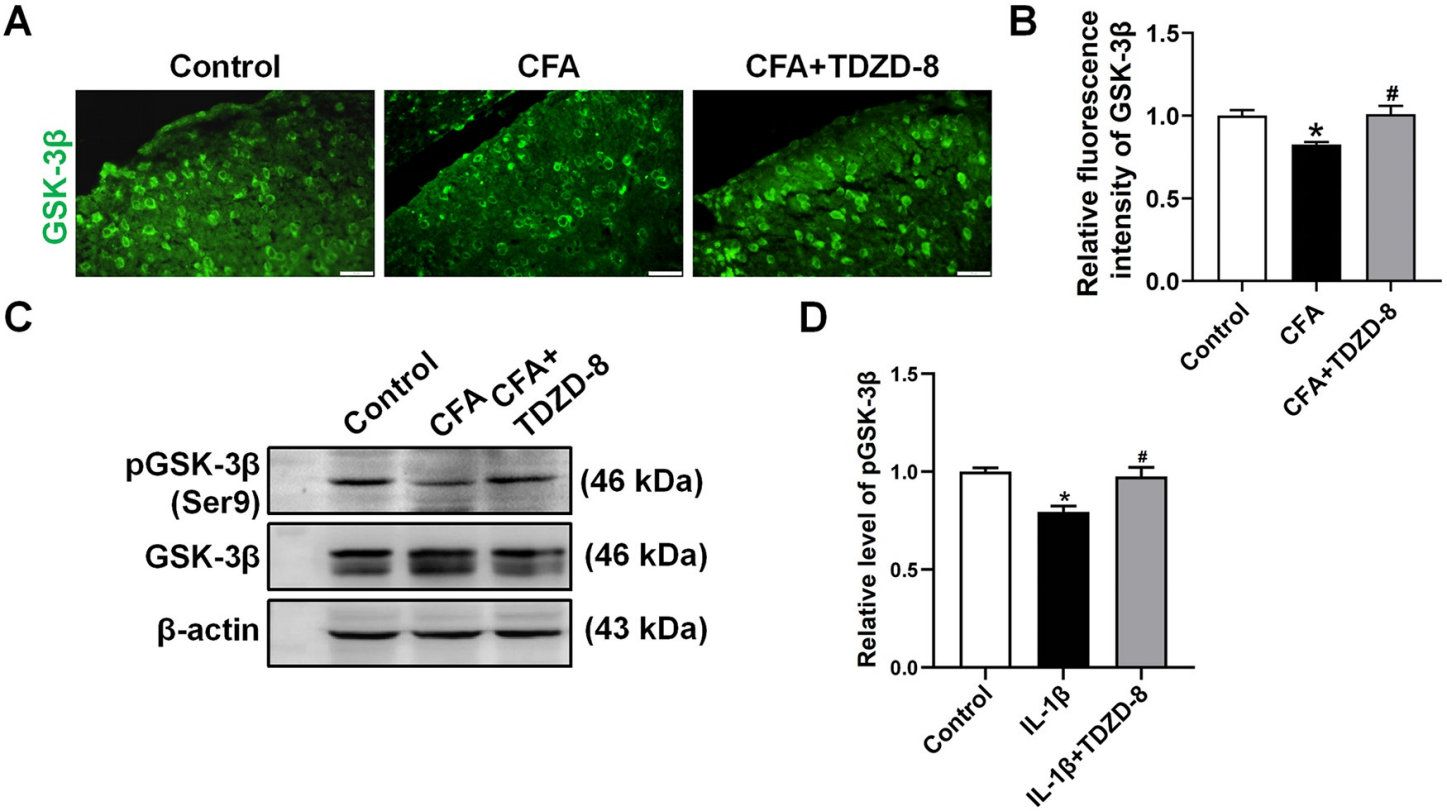
Effect of TDZD-8 treatment on GSK-3β activity. (A, B) Representative immunofluorescence staining images (A) and quantitative intensity analysis of GSK-3β in spinal dorsal horn of control, CFA and CFA + TDZD-8 groups (B). Scale bar = 20 μm. (C, D) Western blot analysis and relative grey values of expression levels of phosphorylated GSK-3β (Ser9) in spinal cord. The relative level of the phosphorylated GSK-3β (Ser9) was normalized to the total GSK-3β. β-actin was used as a loading control. Data are presented as mean ± SD (n = 3). *P < 0.05 *vs*. control group, ^#^*P* < 0.05 *vs*. CFA group.

The effect of TDZD-8 on GSK-3β expression and activity were detected by immunofluorescence assay and Western blot analysis. Since Phosphorylation of GSK-3β at Ser9 presents the inactivity state of GSK-3β [19], we detected the fluorescence intensity of phosphorylated GSK-3β at Ser9. Immunofluorescence assay showed decreased intensity of GSK-3β at Ser9 in spinal dorsal horn of CFA group while TDZD-8 treatment increased the intensity of this site (Fig 3A and 3B). Western blot analysis indicated that phosphorylated GSK-3β at Ser9 level was down-regulated in the CFA group and increased upon TDZD-8 treatment in CFA+TDZD-8 group (Fig 3C and 3D).

### TDZD-8 treatment recovered mitochondrial dysfunction

**Fig 4:**
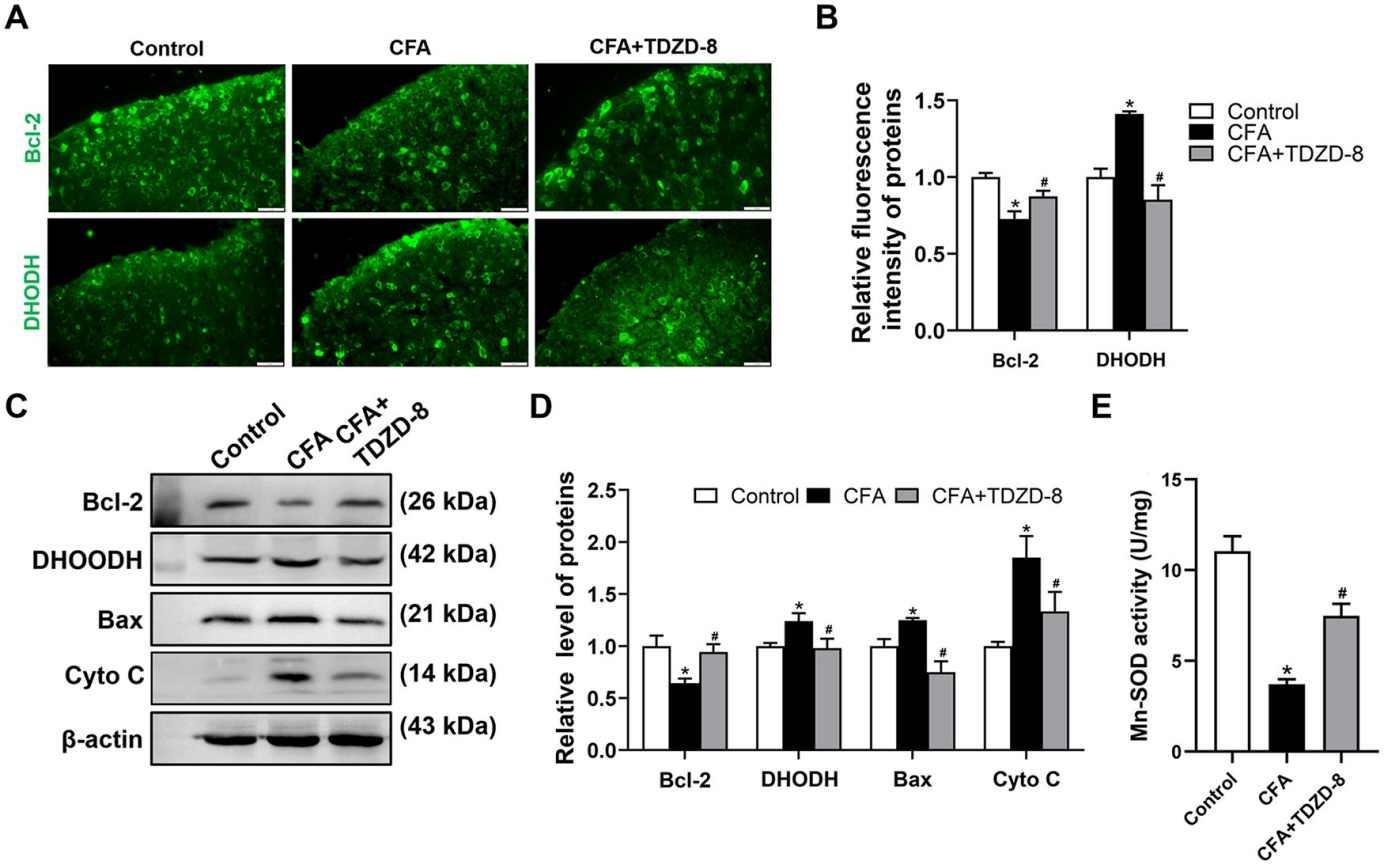
Effect of TDZD-8 treatment on oxidative stress level. (A) Immunofluorescence staining of Bcl-2 and DHODH on spinal cord sections. Scale bar = 20 μm. (B) Quantitative analysis of Bcl-2 and DHODH fluorescence intensity. Data are expressed as the mean ± SD (n = 3). (C, D) Western blot analysis (C) and relative grey values (D) of expression levels of Bcl-2 and DHODH in spinal cord. β-actin was used as a loading control. (E) Changes in Mn-SOD activity detected by Mn-SOD Assay Kit. Data were expressed as the mean ± SD (n = 3). *P < 0.05 *vs*. control group, ^#^*P* < 0.05 *vs*. CFA group.

The expression levels of mitochondrial outer membrane Bax and Bcl-2 were analyzed. In comparison with control group, fluorescence intensity of Bcl-2 in spinal dorsal horn of CFA group was significantly weakened (Fig 4A and 4B), and expression level of spinal Bcl-2 was obviously reduced in CFA group. Reversely, Bax expression level was elevated in CFA group (Fig 4C and 4D). Mitochondrial enzymes DHODH expression and Mn-SOD activity was further detected. The images showed that the fluorescence intensity of DHODH in spinal dorsal horn of CFA group were significantly enhanced (Fig 4A and 4B). Presenting bands for expression levels of spinal DHODH was up-regulated in CFA group (Fig 4C and 4D). While, TDZD-8 treatment statistically decreased the fluorescence intensity of DHODH (Fig 4A and 4B) and reduced grey values of spinal DHODH in CFA group (Fig 4C and 4D). Mn-SOD activity, the primary antioxidant enzyme in mitochondria, dramatically decreased in spinal cord of CFA mice. TDZD-8 treatment remarkably increased Mn-SOD activity of CFA mice, and had no effect on control mice (Fig 4E). Moreover, Cyto C expression was up-regulated in CFA group, and TDZD-8 treatment down-regulated Cyto C expression level.

### TDZD-8 reduces IL-1β induced mitochondrial ROS levels in cells

**Fig 5:**
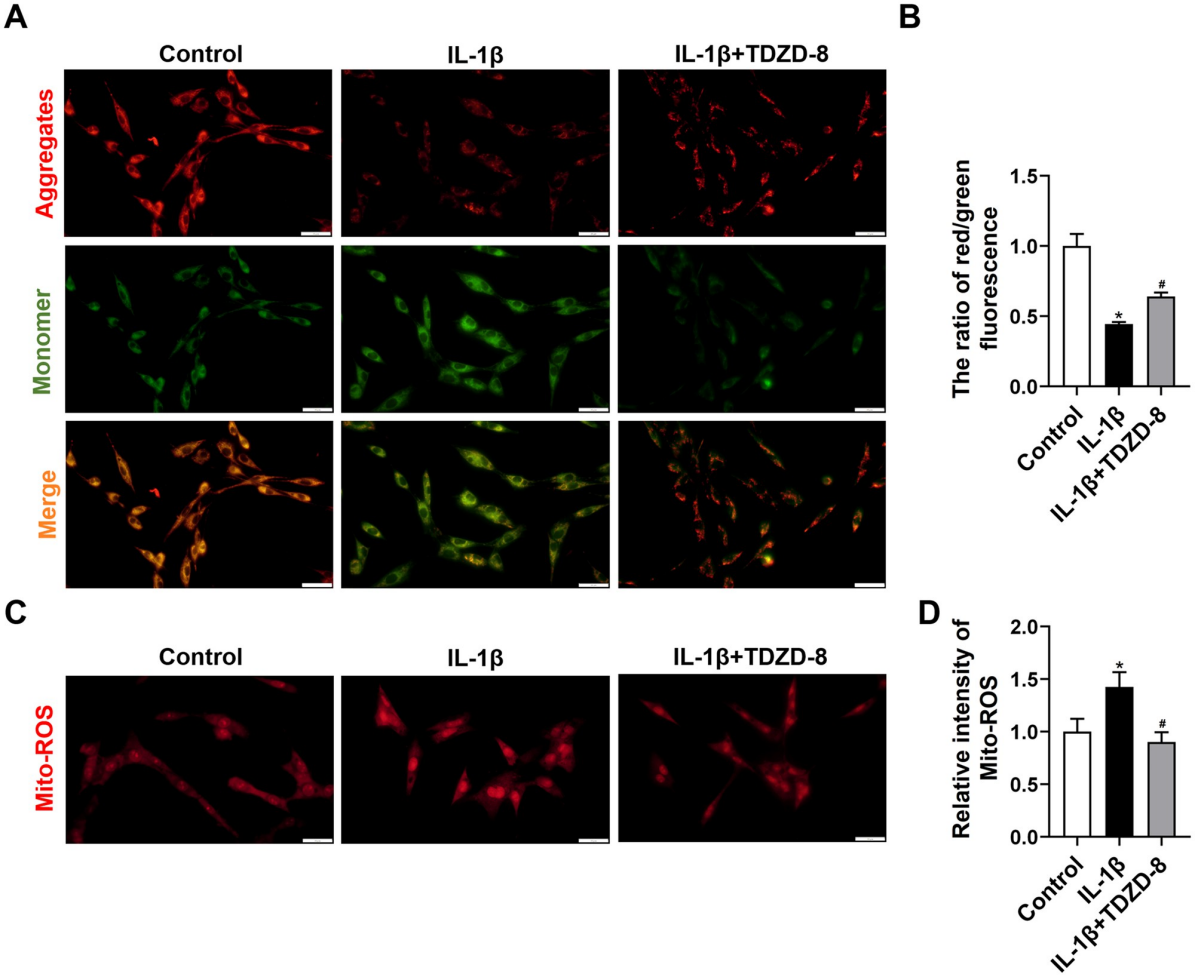
Effect of TDZD-8 on mitochondrial function in IL-1β-induced C6 cells. (A) Detection of the mitochondrial membrane potential in control, IL-1β, and IL-1β + TDZD-8 groups by labeling with the JC-1. Scale bar = 20 μm. (C) Detection of mitochondrial ROS by labeling with the MitoSOX. Scale bar = 20 μm. (B, D) Quantitative fluorescence intensity of JC-1 (C) and Mito-ROS (D). Data are expressed as the mean ± SD. *P < 0.05 *vs*. control group, ^#^*P* < 0.05 *vs*. IL-1β group.

The changes of mitochondrial membrane potential (MMP) were detected by JC-1. Monomeric green form presents lower MMP, while red aggregates form presents higher MMP. IL-1β stimulation in C6 cells induced lower MMP. TDZD-8 treatment restored MMP in IL-1β+TDZD-8 group (Fig 5A and 5B). Mitochondrial ROS level was detected using MitoSOX red indicator which specifically bind with mitochondrial superoxide. Mito-ROS intensity was enhanced after IL-1β stimulation, and TDZD-8 treatment obviously reduced mito-ROS level in IL-1β+TDZD-8 group (Fig 5C and 5D).

## Discussion

GSK-3β participates arthritis pain via modulating mitochondrial mediated oxidative stress. Mitochondrial distribution and morphology depend on energy requirements. High energy requirements in neuron drives mitochondrial fragment and dysfunction, which causes mitochondrial ROS releasing into the cytoplasm [29, 30]. Complexes I, II and III of electron transport chain have been identified as the most relevant sites of ROS production [31]. Complex III transfers the electrons received from complex I and complex II to Cyto C [32]. During these processes, electron leaks and interacts with O_2_ to the generation of O_2_^•–^, which is converted into H_2_O_2_ by Mn-SOD [14]. While, a block in electron transfer across complex III induces ROS production [33]. DHODH associates with respiratory complexes II and III and mediated ROS generation. Bax is a member of the Bcl-2 protein family, resides in the outer mitochondrial membrane, and controls mitochondrial permeability [34]. Activated Bax increases mitochondrial permeability, disrupts MMP and ATP depletion, induces Cyto C release, promoted caspase-3 and -7, following by both activating caspase-8 and IL-1β maturation and secretion [35–37]. While, Bcl-2 prevents Bax oligomerization in the mitochondrial outer membrane [38]. In our study, CFA inducement increased DHODH and Bax activity, reduced Bcl-2 and Mn-SOD activity. GSK-3β regulates mitochondrial function via several ways. GSK-3β inhibits the activity of complex I-IV in the respiratory chain, attenuates ATP production and produces more ROS [39]. Activated GSK-3β directly interacted with Bax, caused Bax oligomerization and activation [28]. GSK-3β modulates mitochondrial biogenesis by regulating the expression of peroxisome proliferator-activated receptor gamma coactivator 1 alpha (PGC-1α) [40]. GSK-3β is reported to phosphorylate mitochondrial transporter dynein at Ser87 and Thr88, negatively regulates dynein and mitochondrial motility [41]. In our study, we found GSK-3β inhibition decreased DHODH and Bax expression, and increased Mn-SOD activity, reduced mitochondrial mediated oxidative stress.

GSK-3β regulates inflammation via modulating NF-κB recruitment and regulating gene transcription. NF-κB served as a pivotal mediator of inflammatory responses via inducing the expression of various pro-inflammatory genes, including those encoding cytokines and chemokines, and also participating in NLRP3 inflammasome regulation [42, 43]. In GSK-3β null cells or cells treated with a GSK-3β pharmacological inhibitor, NF-κB DNA binding activity is reduced [44]. GSK-3β also modulated NF-κB essential modifier NEMO phosphorylation at serine 8, 17, 31 and 43, and ordered NF-κB signaling [45]. In our study, we found GSK-3β inhibition decreased NF-κB expression and the activation of NLRP3 inflammation.

## Conclusion

**Fig 6:**
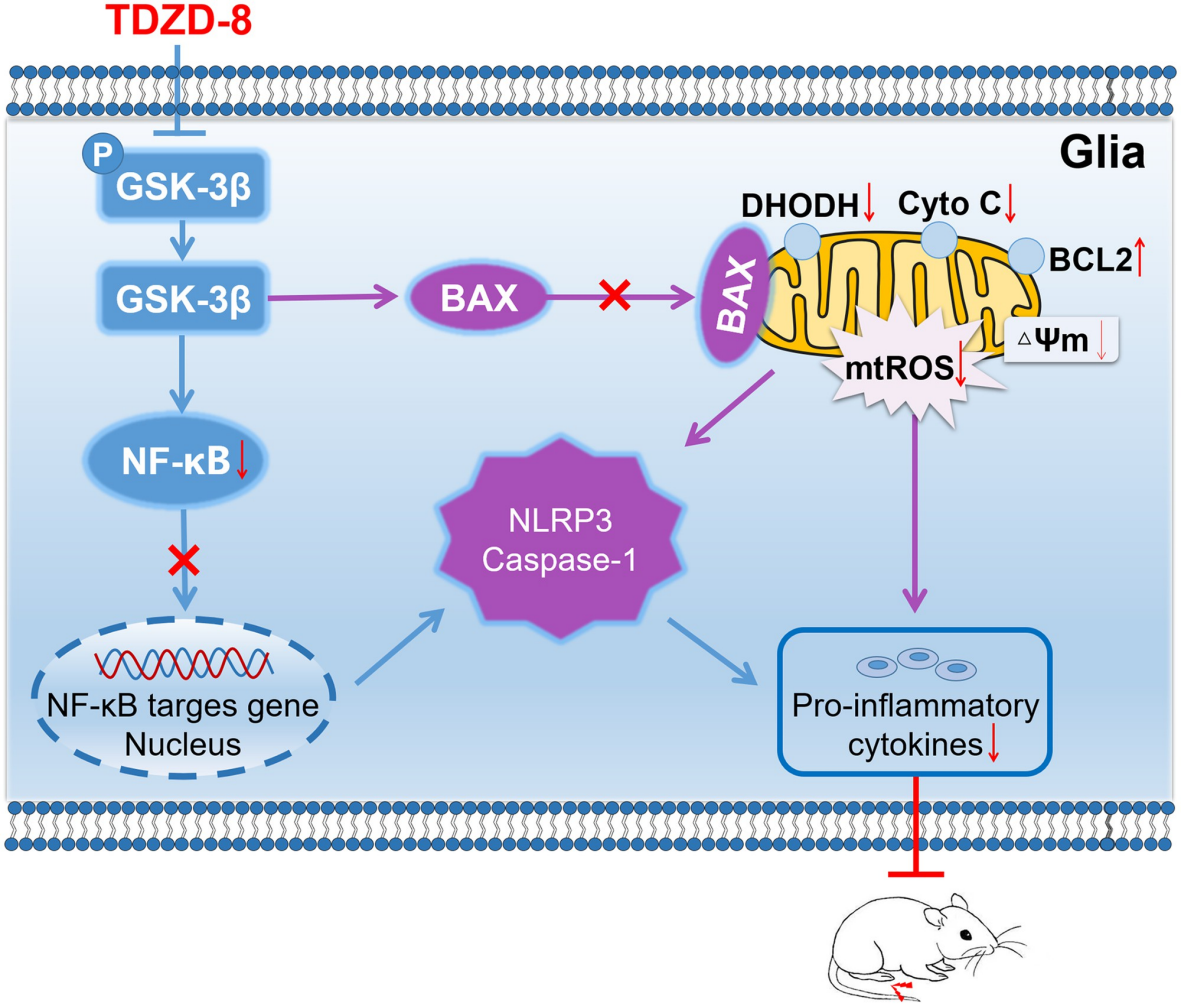
Schematic representation of the potential mechanisms of TDZD-8 treatment on alleviating arthritis pain.

During osteoarthritis processing, spinal inflammatory reaction is triggered, mitochondrial-mediated oxidative stress is increased, and neuropathic pain is triggered. GSK-3β inhibitor TDZD-8 treatment suppresses spinal Bax and DHODH mediated mitochondrial ROS levels, inhibits NF-κB mediated NLRP3 inflammation activation, and alleviates arthritis pain (Fig 6).

## Supporting information

S1 Raw images (PDF)

## References

[1] Hunter DJ, Bierma-Zeinstra S. Osteoarthritis. Lancet. 2019;393(10182):1745–1759. doi: 10.1016/S0140-6736(19)30417-9 31034380

[2] Yu H, Huang T, Lu WW, Tong L, Chen D. Osteoarthritis Pain. Int J Mol Sci. 2022;23(9):4642. doi: 10.3390/ijms23094642 35563035 PMC9105801

[3] Crow JA, Fillingim RB. Working toward mechanistic pain phenotyping in osteoarthritis. Osteoarthritis Cartilage. 2022;30(4):495–497. doi: 10.1016/j.joca.2021.11.018 34875376

[4] Trouvin AP, Perrot S. Pain in osteoarthritis. Implications for optimal management. Joint Bone Spine. 2018;85(4):429–434. doi: 10.1016/j.jbspin.2017.08.002 28889010

[5] Pan TT, Pan F, Gao W, Hu SS, Wang D. Involvement of Macrophages and Spinal Microglia in Osteoarthritis Pain. Curr Rheumatol Rep. 2021;23(5):29. doi: 10.1007/s11926-021-00997-w 33893883

[6] Fayet M, Hagen M. Pain characteristics and biomarkers in treatment approaches for osteoarthritis pain. Pain Manag. 2021;11(1):59–73. doi: 10.2217/pmt-2020-0055 33124514

[7] Bajaj P, Bajaj P, Graven-Nielsen T, Arendt-Nielsen L. Osteoarthritis and its association with muscle hyperalgesia: an experimental controlled study. Pain. 2001;93(2):107–114. doi: 10.1016/S0304-3959(01)00300-1 11427321

[8] French HP, Smart KM, Doyle F. Prevalence of neuropathic pain in knee or hip osteoarthritis: A systematic review and meta-analysis. Semin Arthritis Rheum. 2017;47(1):1–8. doi: 10.1016/j.semarthrit.2017.02.008 28320529

[9] Zhao F, Williams M, Meng X, Welsh DC, Grachev ID, Hargreaves R, et al. Pain fMRI in rat cervical spinal cord: an echo planar imaging evaluation of sensitivity of BOLD and blood volume-weighted fMRI. Neuroimage. 2009;44(2):349–362. doi: 10.1016/j.neuroimage.2008.09.001 18835453

[10] Ji RR, Nackley A, Huh Y, Terrando N, Maixner W. Neuroinflammation and Central Sensitization in Chronic and Widespread Pain. Anesthesiology. 2018;129(2):343–366. doi: 10.1097/ALN.0000000000002130 29462012 PMC6051899

[11] Sagar DR, Burston JJ, Hathway GJ, Woodhams SG, Pearson RG, Bennett AJ, et al. The contribution of spinal glial cells to chronic pain behaviour in the monosodium iodoacetate model of osteoarthritic pain. Mol Pain. 2011;7:88. doi: 10.1186/1744-8069-7-88 22093915 PMC3271989

[12] Adães S, Almeida L, Potes CS, Ferreira AR, Castro-Lopes JM, Ferreira-Gomes J, et al. Glial activation in the collagenase model of nociception associated with osteoarthritis. Mol Pain. 2017;13:1744806916688219. doi: 10.1177/1744806916688219 28326927 PMC5302176

[13] Bruehl S, Milne G, Schildcrout J, Shi Y, Anderson S, Shinar A, et al. Oxidative stress is associated with characteristic features of the dysfunctional chronic pain phenotype. Pain. 2022;163(4):786–794. doi: 10.1097/j.pain.0000000000002429 34382610 PMC8807797

[14] Raimondi V, Ciccarese F, Ciminale V. Oncogenic pathways and the electron transport chain: a dangeROS liaison. Br J Cancer. 2020;122(2):168–181. doi: 10.1038/s41416-019-0651-y 31819197 PMC7052168

[15] Chen L, Tian Q, Shi Z, Qiu Y, Lu Q, Liu C. Melatonin Alleviates Cardiac Function in Sepsis-Caused Myocarditis via Maintenance of Mitochondrial Function. Front Nutr. 2021;8:754235. doi: 10.3389/fnut.2021.754235 34708067 PMC8542660

[16] Chen L, Tian Q, Shi Z, Qiu Y, Lu Q, Liu C. Sestrin2 overexpression attenuates osteoarthritis pain via induction of AMPK/PGC-1α-mediated mitochondrial biogenesis and suppression of neuroinflammation. Brain Behav Immun. 2022;102:53–70. doi: 10.1016/j.bbi.2022.02.015 35151829

[17] Lagos-Rodríguez V, Martínez-Palma L, Marton S, Miquel E, Escobar-Pintos R, Cassina A, et al. Mitochondrial bioenergetics, glial reactivity, and pain-related behavior can be restored by dichloroacetate treatment in rodent pain models. Pain. 2020;161(12):2786–2797. doi: 10.1097/j.pain.0000000000001992 32658145

[18] Chen XJ, Wang L, Song XY. Mitoquinone alleviates vincristine-induced neuropathic pain through inhibiting oxidative stress and apoptosis via the improvement of mitochondrial dysfunction. Biomed Pharmacother. 2020;125:110003. doi: 10.1016/j.biopha.2020.110003 32187955

[19] Rashvand M, Danyali S, Manaheji H. The Potential Role of Glycogen Synthase Kinase-3β in Neuropathy-Induced Apoptosis in Spinal Cord. Basic Clin Neurosci. 2020;11(1):15–30. doi: 10.32598/bcn.11.1.1 32483472 PMC7253818

[20] Peng Z, Zha L, Yang M, Li Y, Guo X, Feng Z. Effects of ghrelin on pGSK-3β and β-catenin expression when protects against neuropathic pain behavior in rats challenged with chronic constriction injury. Sci Rep. 2019;9(1):14664. doi: 10.1038/s41598-019-51140-w 31601982 PMC6787073

[21] Yang HY, Zhang F, Cheng ML, Wu J, Xie M, Yu LZ, et al. Glycogen synthase kinase-3β inhibition decreases inflammation and relieves cancer induced bone pain via reducing Drp1-mediated mitochondrial damage. J Cell Mol Med. 2022;26(14):3965–3976. doi: 10.1111/jcmm.17432 35689386 PMC9279596

[22] Shu Z, Miao X, Tang T, Zhan P, Zeng L, Jiang Y. The GSK-3β/β-catenin signaling pathway is involved in HMGB1-induced chondrocyte apoptosis and cartilage matrix degradation. Int J Mol Med. 2020;45(3):769–778. doi: 10.3892/ijmm.2020.4460 31922219 PMC7015138

[23] Torres-Guzman AM, Morado-Urbina CE, Alvarado-Vazquez PA, Acosta-Gonzalez RI, Chávez-Piña AE, Montiel-Ruiz RM, et al. Chronic oral or intraarticular administration of docosahexaenoic acid reduces nociception and knee edema and improves functional outcomes in a mouse model of Complete Freund’s Adjuvant-induced knee arthritis. Arthritis Res Ther. 2014;16(2):R64. doi: 10.1186/ar4502 24612981 PMC4060174

[24] Cuzzocrea S, Crisafulli C, Mazzon E, Esposito E, Muià C, Abdelrahman M, et al. Inhibition of glycogen synthase kinase-3beta attenuates the development of carrageenan-induced lung injury in mice. Br J Pharmacol. 2006;149(6):687–702. doi: 10.1038/sj.bjp.0706902 17016509 PMC2014652

[25] Hao M, Tang Q, Wang B, Li Y, Ding J, Li M, et al. Resveratrol suppresses bone cancer pain in rats by attenuating inflammatory responses through the AMPK/Drp1 signaling. Acta Biochim Biophys Sin (Shanghai). 2020;52(3):231–240. doi: 10.1093/abbs/gmz162 32072182

[26] Mao Y, Wang C, Tian X, Huang Y, Zhang Y, Wu H, et al. Endoplasmic Reticulum Stress Contributes to Nociception via Neuroinflammation in a Murine Bone Cancer Pain Model. Anesthesiology. 2020;132(2):357–372. doi: 10.1097/ALN.0000000000003078 31939851

[27] Shi X, Bai H, Wang J, Wang J, Huang L, He M, et al. Behavioral Assessment of Sensory, Motor, Emotion, and Cognition in Rodent Models of Intracerebral Hemorrhage. Front Neurol. 2021;12:667511. doi: 10.3389/fneur.2021.667511 34220676 PMC8248664

[28] Liu Y, Huang Y, Ding J, Liu N, Peng S, Wang J, et al. Targeting Akt by SC66 triggers GSK-3β mediated apoptosis in colon cancer therapy. Cancer Cell Int. 2019;19:124. doi: 10.1186/s12935-019-0837-7 31168297 PMC6509835

[29] Faria-Pereira A, Morais VA. Synapses: The Brain’s Energy-Demanding Sites. Int J Mol Sci. 2022;23(7):3627. doi: 10.3390/ijms23073627 35408993 PMC8998888

[30] Li MY, Ding JQ, Tang Q, Hao MM, Wang BH, Wu J, et al. SIRT1 activation by SRT1720 attenuates bone cancer pain via preventing Drp1-mediated mitochondrial fission. Biochim Biophys Acta Mol Basis Dis. 2019;1865(3):587–598. doi: 10.1016/j.bbadis.2018.12.017 30579931

[31] Nolfi-Donegan D, Braganza A, Shiva S. Mitochondrial electron transport chain: Oxidative phosphorylation, oxidant production, and methods of measurement. edox Biol. 2020;37:101674. doi: 10.1016/j.redox.2020.101674 32811789 PMC7767752

[32] Mazat JP, Devin A, Ransac S. Modelling mitochondrial ROS production by the respiratory chain. Cell Mol Life Sci. 2020;77(3):455–465. doi: 10.1007/s00018-019-03381-1 31748915 PMC11104992

[33] Quinlan CL, Gerencser AA, Treberg JR, Brand MD. The mechanism of superoxide production by the antimycin-inhibited mitochondrial Q-cycle. J Biol Chem. 2011;286(36):31361–31372. doi: 10.1074/jbc.M111.267898 21708945 PMC3173136

[34] Gao W, Hu L, Zhang M, Liu S, Xu S, Chow VL, et al. Mitochondrial DHODH regulates hypoxia-inducible factor 1 expression in OTSCC. Am J Cancer Res. 2022;12(1):48–67. 35141004 PMC8822278

[35] Vince JE, De Nardo D, Gao W, Vince AJ, Hall C, McArthur K, et al. The Mitochondrial Apoptotic Effectors BAX/BAK Activate Caspase-3 and -7 to Trigger NLRP3 Inflammasome and Caspase-8 Driven IL-1β Activation. Cell Rep. 2018;25(9):2339–2353.e4. doi: 10.1016/j.celrep.2018.10.103 30485804

[36] Slevin E, Baiocchi L, Wu N, Ekser B, Sato K, Lin E, et al. Kupffer Cells: Inflammation Pathways and Cell-Cell Interactions in Alcohol-Associated Liver Disease. Am J Pathol. 2020;190(11):2185–2193. doi: 10.1016/j.ajpath.2020.08.014 32919978 PMC7587925

[37] Dong ZB, Wang YJ, Cheng ML, Wang BJ, Lu H, Zhu HL, et al. 2-Bromopalmitate decreases spinal inflammation and attenuates oxaliplatin-induced neuropathic pain via reducing Drp1-mediated mitochondrial dysfunction. PLoS One. 2022;17(10):e0275428. doi: 10.1371/journal.pone.0275428 36315519 PMC9621438

[38] Shi CS, Kehrl JH. Bcl-2 regulates pyroptosis and necroptosis by targeting BH3-like domains in GSDMD and MLKL. Cell Death Discov. 2019;5:151. doi: 10.1038/s41420-019-0230-2 31839993 PMC6901440

[39] Tang X, Ma S, Li Y, Sun Y, Zhang K, Zhou Q, et al. Evaluating the Activity of Sodium Butyrate to Prevent Osteoporosis in Rats by Promoting Osteal GSK-3β/Nrf2 Signaling and Mitochondrial Function. J Agric Food Chem. 2020;68(24):6588–6603. doi: 10.1021/acs.jafc.0c01820 32459091

[40] Yang K, Chen Z, Gao J, Shi W, Li L, Jiang S, et al. The Key Roles of GSK-3β in Regulating Mitochondrial Activity. Cell Physiol Biochem. 2017;44(4):1445–1459. doi: 10.1159/000485580 29190615

[41] Gao FJ, Hebbar S, Gao XA, Alexander M, Pandey JP, Walla MD, et al. GSK-3β Phosphorylation of Cytoplasmic Dynein Reduces Ndel1 Binding to Intermediate Chains and Alters Dynein Motility. Traffic. 2015;16(9):941–961. doi: 10.1111/tra.12304 26010407 PMC4543430

[42] Peng L, Wen L, Shi QF, Gao F, Huang B, Meng J, et al. Scutellarin ameliorates pulmonary fibrosis through inhibiting NF-κB/NLRP3-mediated epithelial-mesenchymal transition and inflammation. Cell Death Dis. 2020;11(11):978. doi: 10.1038/s41419-020-03178-2 33188176 PMC7666141

[43] Dong ZB, Wang YJ, Wan WJ, Wu J, Wang BJ, Zhu HL, et al. Resveratrol ameliorates oxaliplatin-induced neuropathic pain via anti-inflammatory effects in rats. Exp Ther Med. 2022;24(3):586. doi: 10.3892/etm.2022.11523 35949346 PMC9353538

[44] Arab HH, Safar MM, Shahin NN. Targeting ROS-Dependent AKT/GSK-3β/NF-κB and DJ-1/Nrf2 Pathways by Dapagliflozin Attenuates Neuronal Injury and Motor Dysfunction in Rotenone-Induced Parkinson’s Disease Rat Model. ACS Chem Neurosci. 2021;12(4):689–703. doi: 10.1021/acschemneuro.0c00722 33543924

[45] Medunjanin S, Schleithoff L, Fiegehenn C, Weinert S, Zuschratter W, Braun-Dullaeus RC. GSK-3β controls NF-kappaB activity via IKKγ/NEMO. Sci Rep. 2016;6:38553. doi: 10.1038/srep38553 27929056 PMC5144080

