# Supplementary material for: GSK-3β inhibition alleviates arthritis pain via reducing spinal mitochondrial reactive oxygen species level and inflammation
Source: PLoS One. 2023 Apr 14;18(4):e0284332. doi: 10.1371/journal.pone.0284332 (PMC10104309; doi:10.1371/journal.pone.0284332)
Supplement: S1 Raw images — (PDF) [file pone.0284332.s001.pdf]

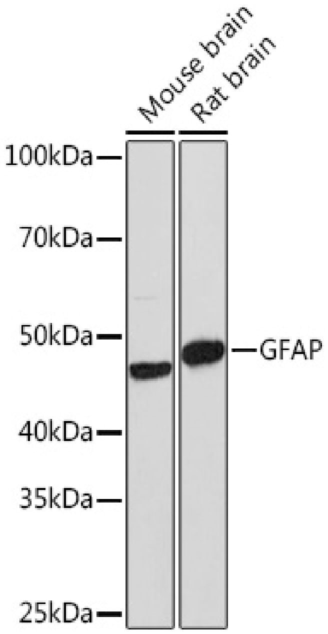


WB bands supplied by ABclonal Technology Co.,Ltd.

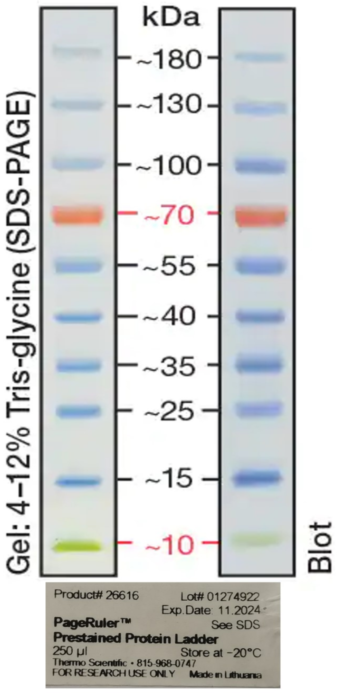


Protein ladder we uesd

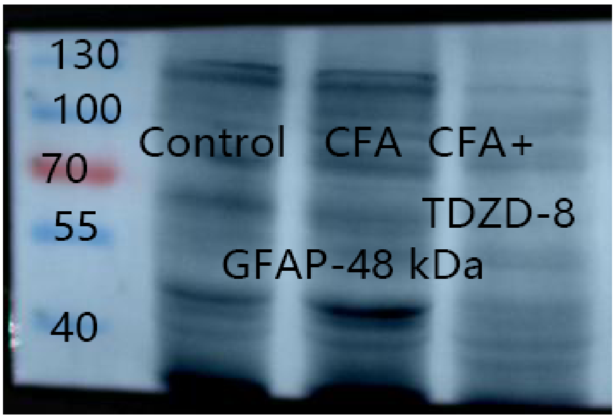


**Raw WB bands for 10% SDS-PAGE**

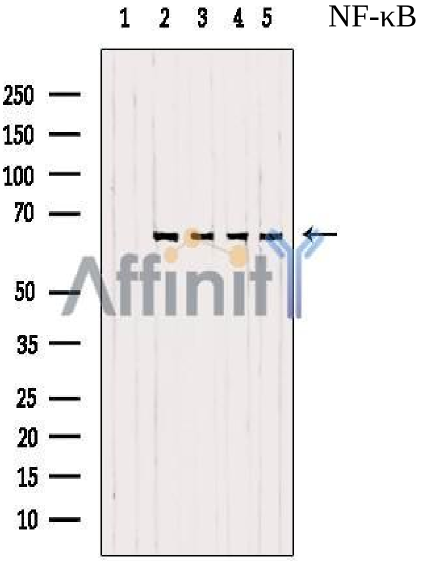


WB bands supplied by Affinity Biosciences

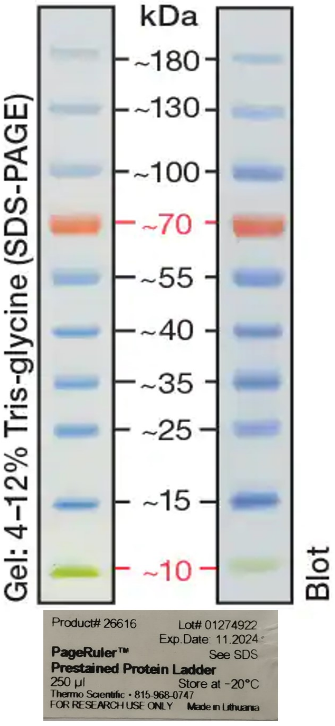


Protein ladder we uesd

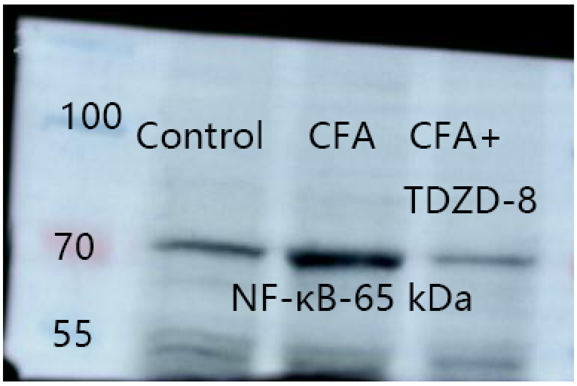


**Raw WB bands for 8% SDS-PAGE**

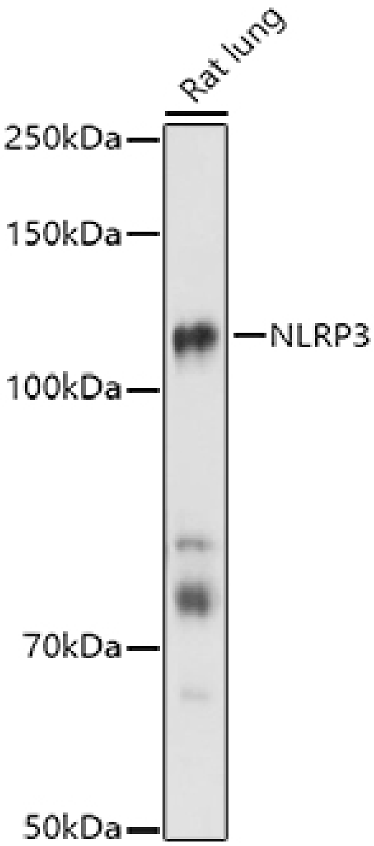


**WB bands supplied by ABclonal Technology Co.,Ltd.**

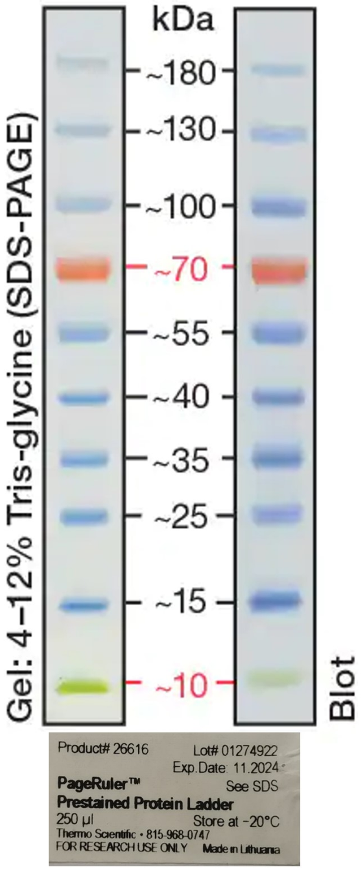


**Protein ladder we uesd**

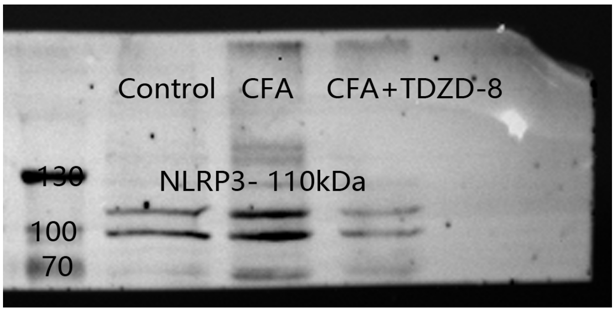


# Raw WB bands for 10% SDS-PAGE

| | |
|---|---|
| 产品应用 | WB IF/ICC |
| 推荐稀释比 | WB 1:500 - 1:2000<br>IF/ICC 1:50 - 1:200 |
| 理论分子量 | 10kDa/29kDa/35kDa/42kDa/45kDa |
| 实际分子量 | 48KDa |

No WB bands supplied by abclone

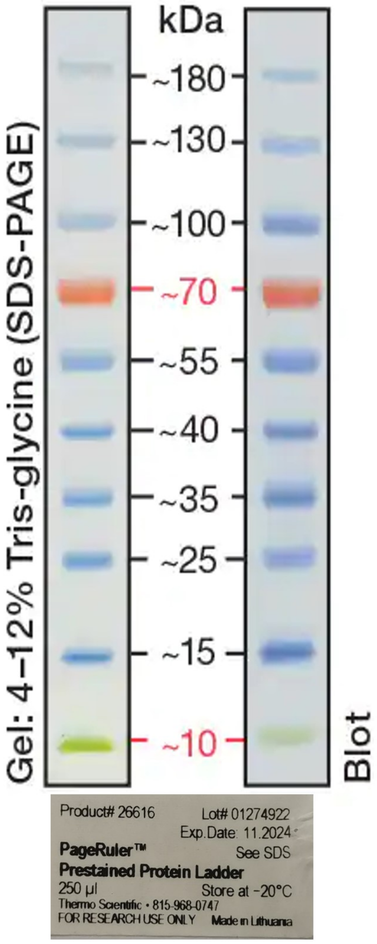


**Protein ladder we uesd**

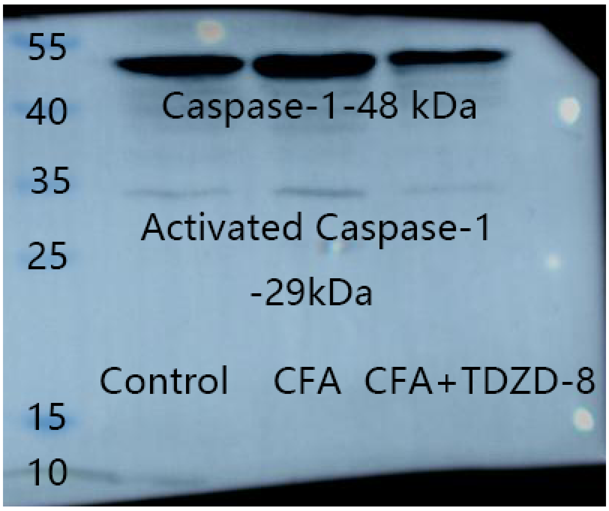


# Raw WB bands for 12% SDS-PAGE

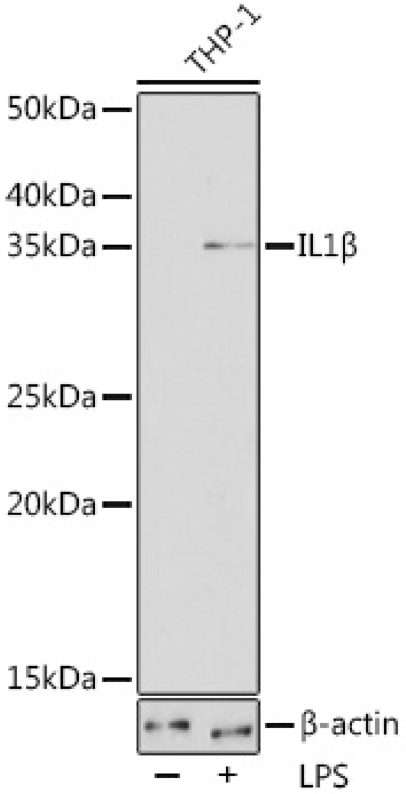


WB bands supplied by ABclonal Technology Co.,Ltd.

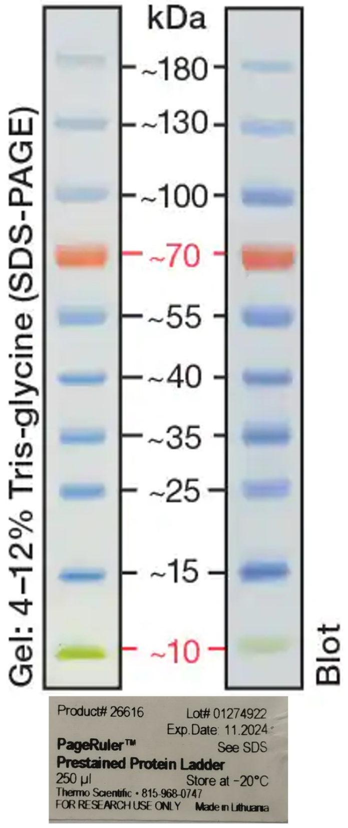


Protein ladder we uesd

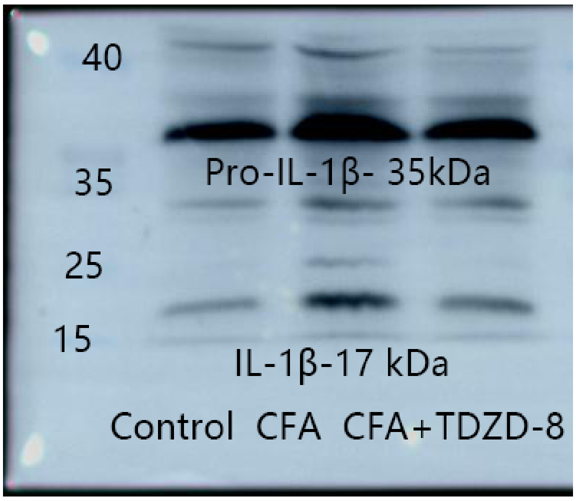


**Raw WB bands for 12% SDS-PAGE**

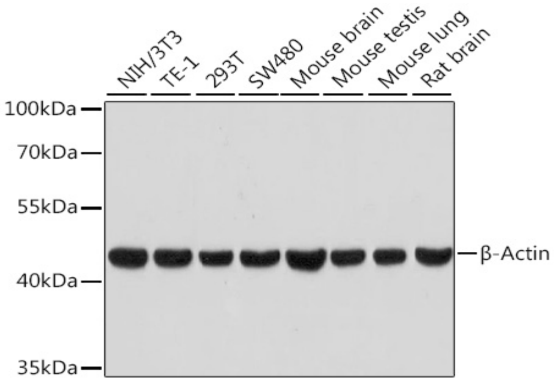


WB bands supplied by ABclonal Technology Co.,Ltd.

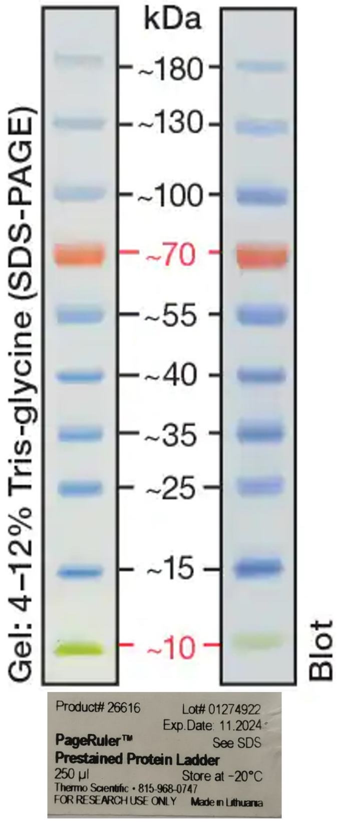


Protein ladder we uesd

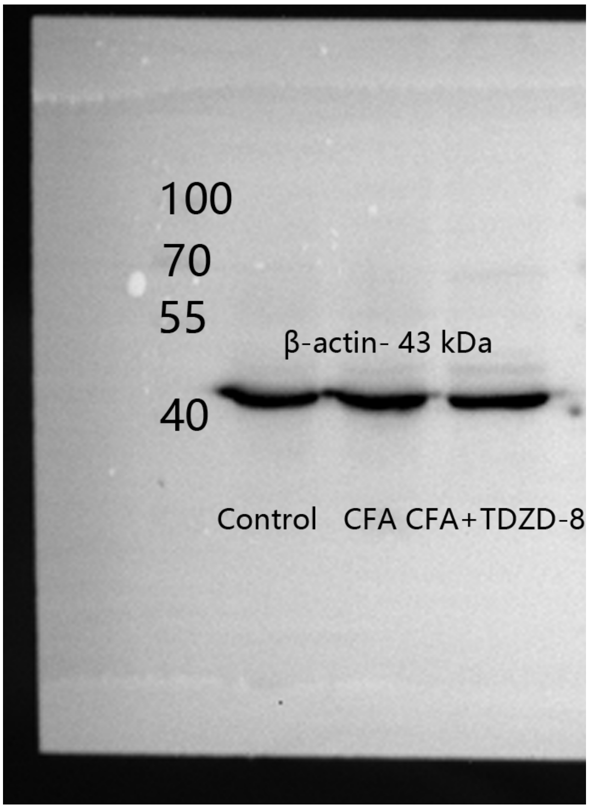


**Raw WB bands for 10% SDS-PAGE**

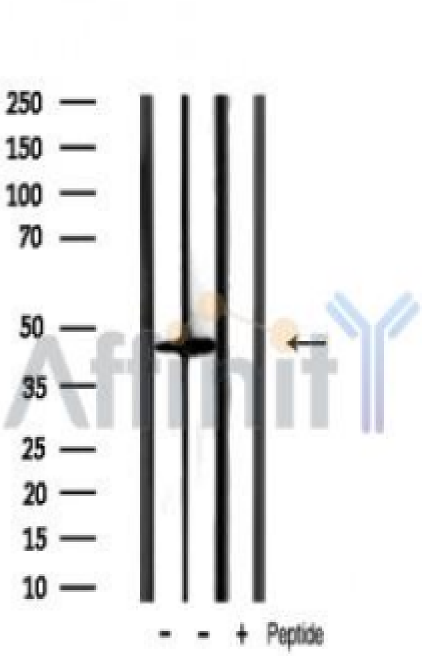


WB bands supplied by ABclonal Technology Co.,Ltd.

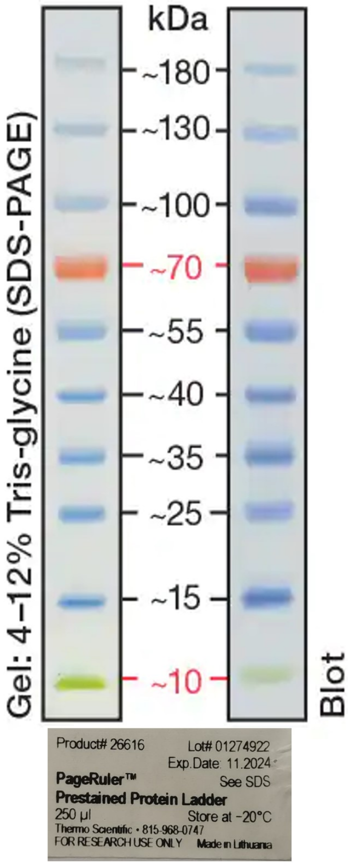


Protein ladder we uesd

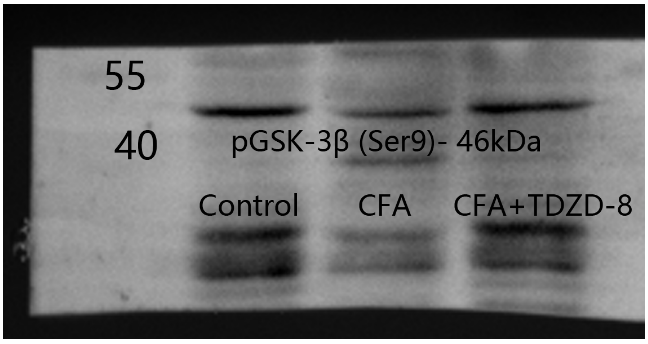


**Raw WB bands for 10% SDS-PAGE**

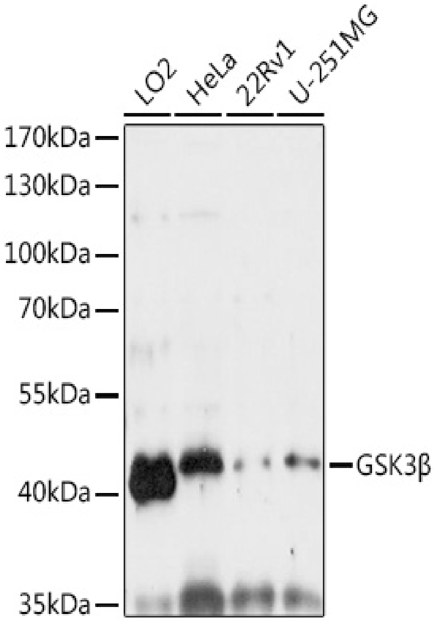


WB bands supplied by ABclonal Technology Co.,Ltd.

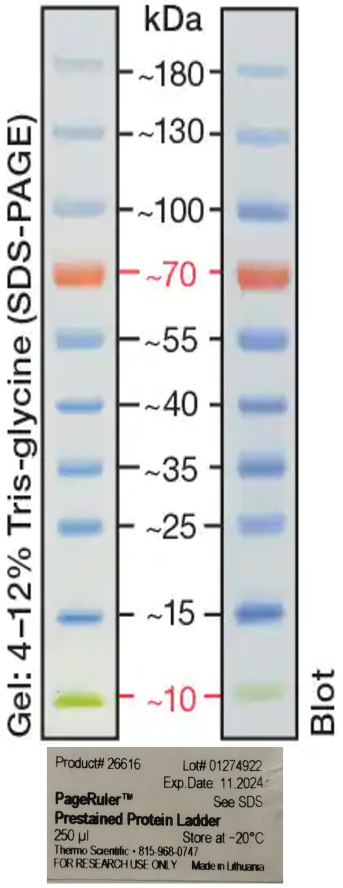


Protein ladder we uesd

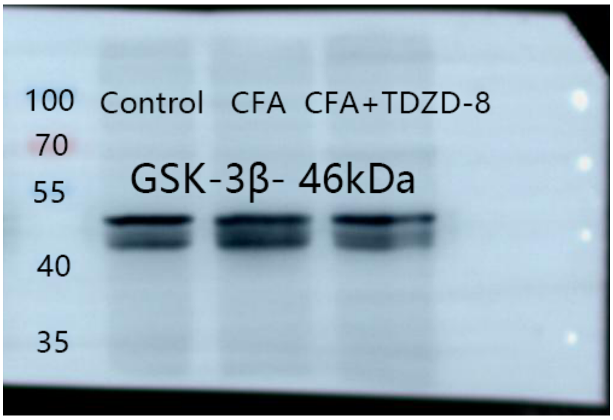


**Raw WB bands for 10% SDS-PAGE**

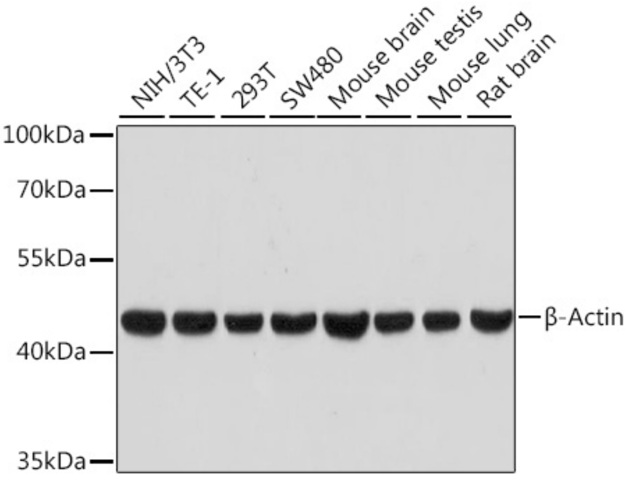


**WB bands supplied by ABclonal Technology Co.,Ltd.**

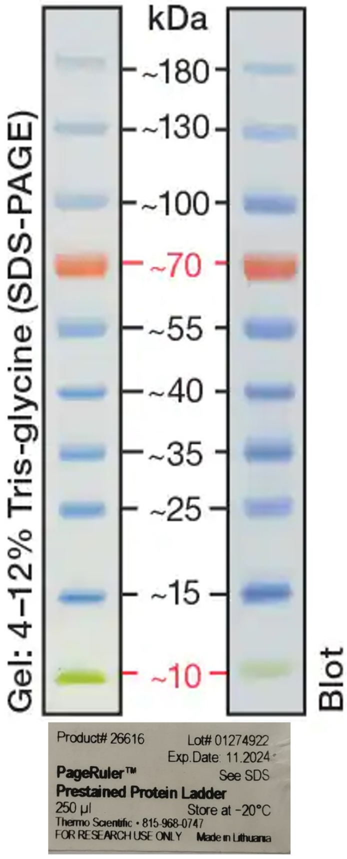


**Protein ladder we uesd**

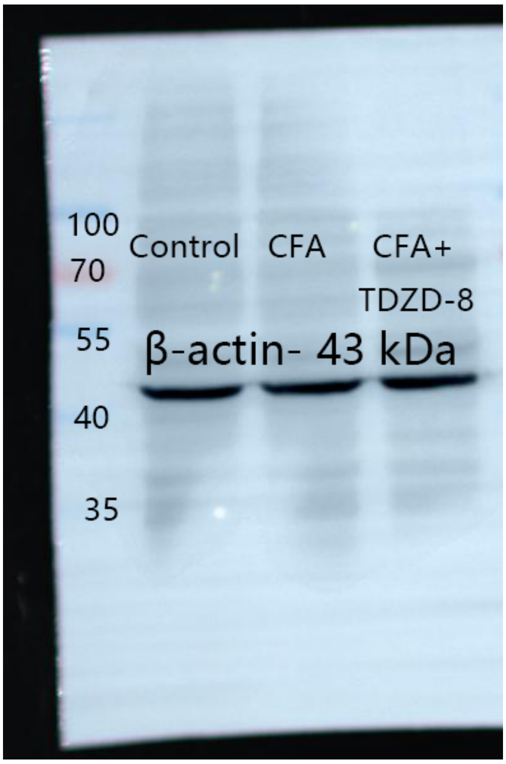


**Raw WB bands for 10% SDS-PAGE**

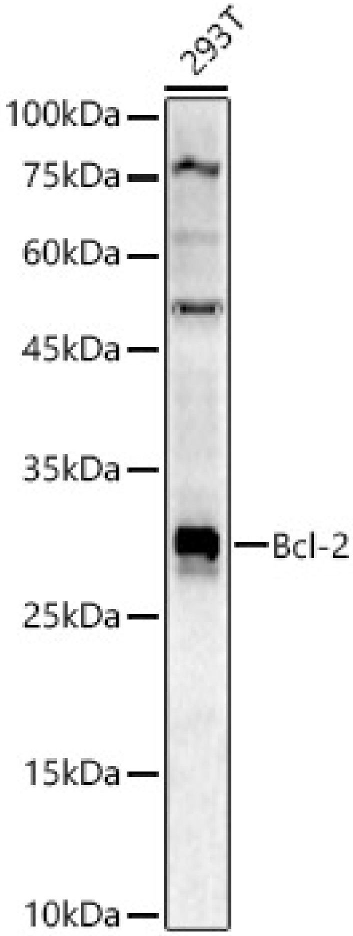


**WB bands supplied by ABclonal Technology Co.,Ltd.**

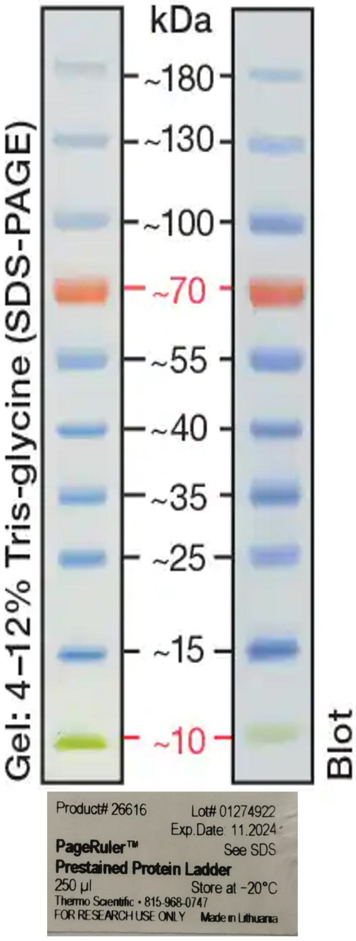


**Protein ladder we uesd**

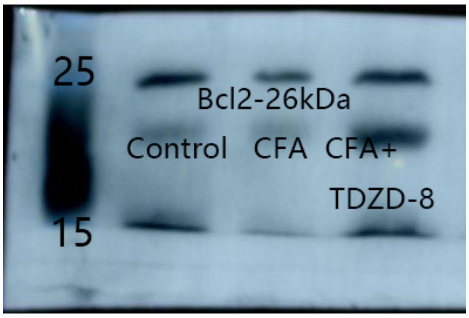


**Raw WB bands for 12% SDS-PAGE**

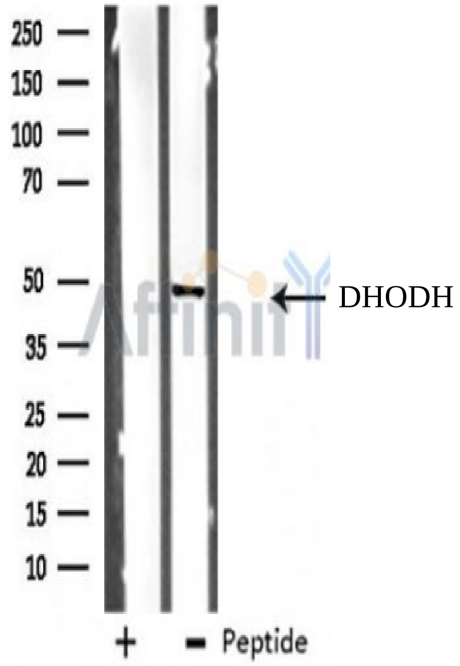


WB bands supplied by Affinity Biosciences

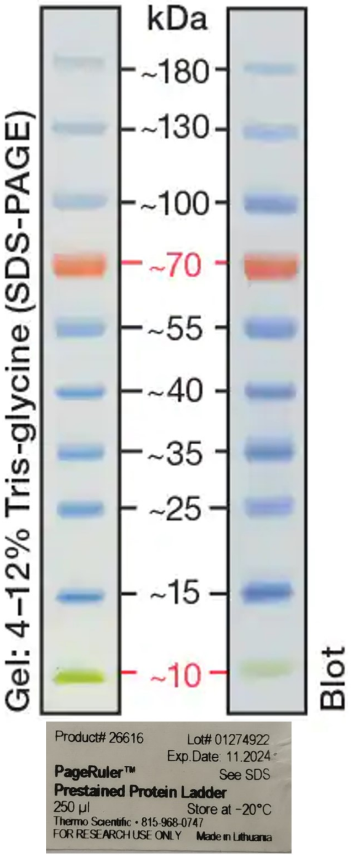


Protein ladder we uesd

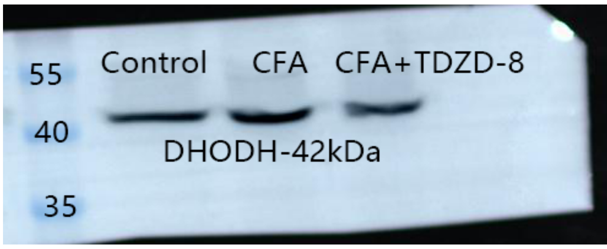


Raw WB bands for 10% SDS-PAGE

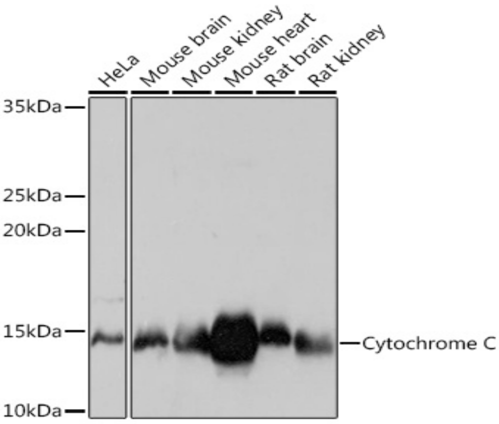


WB bands supplied by ABclonal Technology Co.,Ltd.

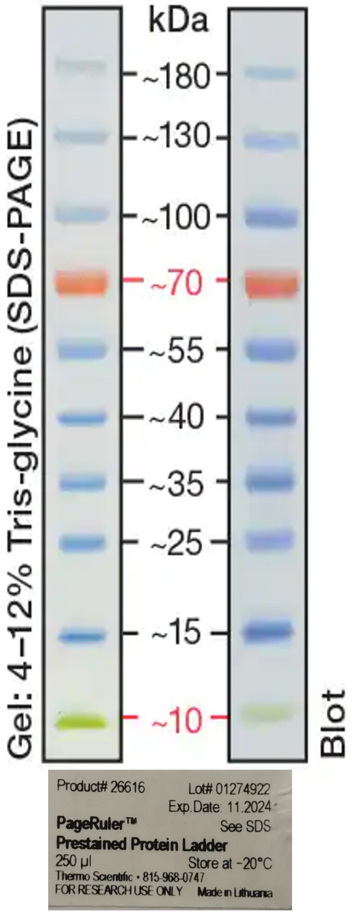


Protein ladder we uesd

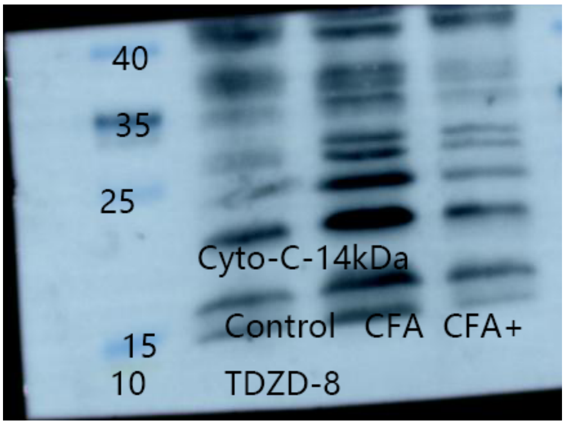


Raw WB bands for 12% SDS-PAGE

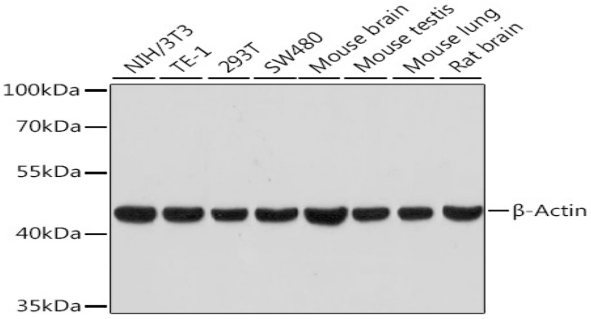


**WB bands supplied by ABclonal Technology Co.,Ltd.**

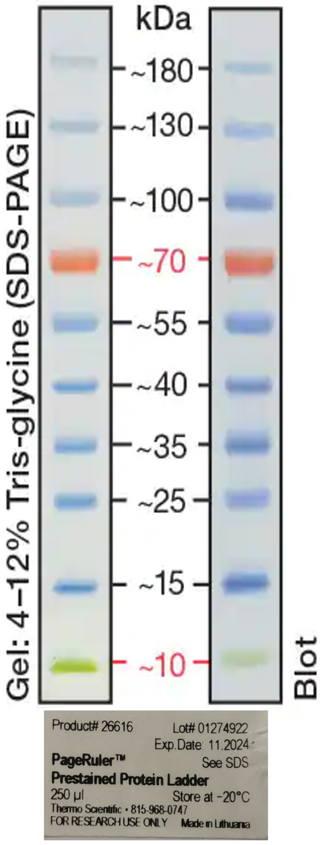


**Protein ladder we uesd**

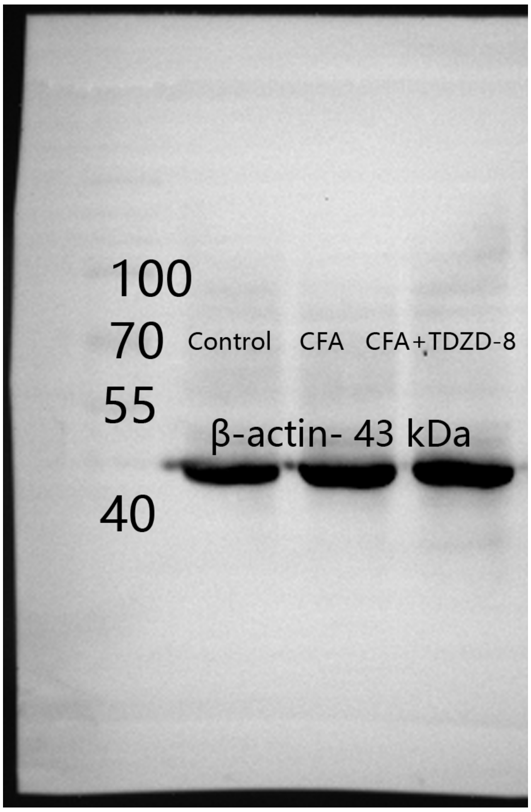


**Raw WB bands for 10% SDS-PAGE**
